# IL-9 and IL-9-producing cells in tumor immunity

**DOI:** 10.1186/s12964-020-00538-5

**Published:** 2020-03-30

**Authors:** Jie Wan, Yinqiu Wu, Xiaoyun Ji, Lan Huang, Wei Cai, Zhaoliang Su, Shengjun Wang, Huaxi Xu

**Affiliations:** 1 Department of Immunology, Jiangsu University, Zhenjiang, 212013 China; 2 China International Genomics Research Center (IGRC), Jiangsu University, Zhenjiang, 212013 China; 3 Department of Laboratory Medicine, The Affiliated People’s Hospital, Jiangsu University, Zhenjiang, 212001 China

**Keywords:** IL-9, Th9, Tc9, Mast cells, Tumor, Tumor diseases

## Abstract

**Abstract:**

Interleukin (IL)-9 belongs to the IL-2Rγc chain family and is a multifunctional cytokine that can regulate the function of many kinds of cells. It was originally identified as a growth factor of T cells and mast cells. In previous studies, IL-9 was mainly involved in the development of allergic diseases, autoimmune diseases and parasite infections. Recently, IL-9, as a double-edged sword in the development of cancers, has attracted extensive attention. Since T-helper 9 (Th9) cell-derived IL-9 was verified to play a powerful antitumor role in solid tumors, an increasing number of researchers have started to pay attention to the role of IL-9-skewed CD8^+^ T (Tc9) cells, mast cells and Vδ2 T cell-derived IL-9 in tumor immunity. Here, we review recent studies on IL-9 and several kinds of IL-9-producing cells in tumor immunity to provide useful insight into tumorigenesis and treatment.

**Video Abstract**

**Graphical abstract:**

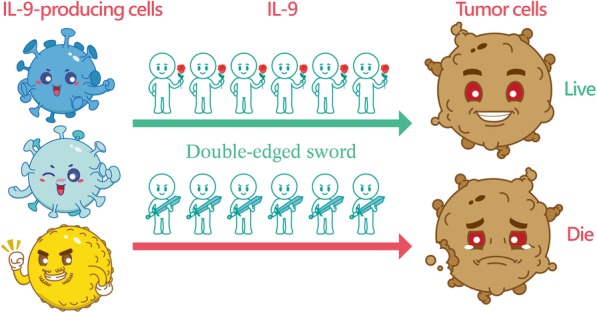

## Background

Every year, there are approximately 11.8 million new cancer cases and 8.4 million deaths worldwide. The overall cancer incidence increases by an average of 3.9% annually. The incidence and mortality rate show an upward trend year by year. Cancer has become one of the four major chronic diseases in the world, seriously affecting people’s health [1]. Currently, effective methods are urgently needed to overcome the development of cancers.

IL-9 was originally identified as a T cell growth factor and is a complex of P40 and TCGF-III, which can be secreted by different cells, such as Th9 cells, type 2 innate lymphoid cells (ILC2s), Tc9 cells, Vδ2 T cells and mast cells [2–6]. CNS-25 is an upstream enhancer of the *Il9* gene, and it can promote the expression of IL-9 when it binds to the most of the *Il9* gene transcription factors [3]. Knockout of CNS-25 can reduce the secretion of IL-9 and weaken the IL-9-dependent immune response [7]. Pleiotropic IL-9 can regulate the function of T cells, B cells, mast cells and airway epithelium cells by activating the STAT1, STAT3 and STAT5 signaling pathways, which are involved in the progression of tumor diseases, allergic diseases, inflammatory, and autoimmune diseases [8]. IL-9 always plays an antitumor role in solid tumors such as melanoma and breast cancer [9–11]. However, in hematological neoplasms such as chronic lymphocytic leukemia, Hodgkin’s lymphoma and diffuse large B lymphoma, IL-9 is generally considered to promote tumor progression via its lymphocyte growth factor function [12–14]. IL-9 can directly affect the survival of tumor cells [15], or indirectly participate in tumor immunity by activating mast cells and recruiting dendritic cells (DCs) into tumor sites [16, 17].

As a new CD4^+^ T-cell subset, Th9 cells were first discovered in 2008 and are characterized by the secretion of the IL-9 cytokine [18]. Th9 cells can mediate tumor immunity and participate in autoimmune diseases or allergic diseases [19, 20]. Previous studies have shown that naive T cells can differentiate into Th9 cells in the presence of IL-4 and TGF-β [21]. In addition, Veldohen et al. demonstrated that TGF-β could promote the transformation of Th2 cells into Th9 cells [22], while Dardalhon et al. demonstrated that IL-4 could block the expression of Foxp3 in Treg cells [23], thus transforming Tregs into a T-cell subset that continuously produces IL-9. Moreover, IL-1β also promotes the differentiation of Th9 cells and the secretion of IL-9 and IL-21. Th9 cell-derived IL-9 and IL-21 can enhance the ability of CD8^+^ T and NKT cells to secrete IFN-γ, thereby promoting tumor killing [24, 25]. Th9 cells were initially studied in allergic diseases and autoimmune diseases [19, 20, 26], and they promote the development of allergic diseases by promoting the expression of the Th2 cell-related chemokines CCL17 and CCL22 [27, 28]. Current studies have shown that Th9 cells play a vital antitumor role in most solid tumors [29]. Th9 cell-mediated antitumor immunity is involved in innate immunity and the adaptive immune response [16, 30]. Th9 cells can promote the secretion of CCL20 from epithelial cells and then induce a potent antitumor CD8^+^ CTL effect by promoting CCL20/CCR6-dependent recruitment of dendritic cells into tumors. CCR6 deficiency may result in loss of the antitumor effect of Th9 cells in mice [16, 31]. Moreover, mast cells play an essential role in Th9 cell-mediated antitumor response [32]. In addition, Th9 cells can directly cause tumor cell death through granzyme B on their surface [33].

CD8^+^ T cells, as important cells involved in the adaptive immune response in antitumor immunity, can be divided into Tc1, Tc2, Tc17 and Tc9 cells [34–36]. Tc9 cells can differentiate from CD8^+^ T cells in a Th9 cell-induced environment [37]. Tc9 cells express very low granzyme B, Eomes, T-bet and IFN-γ which are highly expressed on the typical cytotoxic T cells (CTL), but they can also secrete a large amount of IL-9 [38]. Compared with Tc1 cells, Tc9 cells have a weaker cytolytic ability in vitro, but in OT-I/ B16-OVA and pmel-1/B16 melanoma models, Tc9 cells have a strong and persistent antitumor effect [39, 40]. The antitumor effect of Tc9 cells mainly depends on the production of IL-9 [41]. Cholesterol can negatively regulate the production of IL-9 by Tc9 cells, thus affecting the antitumor immunity [42]. In addition, adoptive transfer of Tc9 cells can produce a strong antitumor effect in the MC38-GP100 tumor model, and this effect could be reversed by anti-IL-9 [38]. The in-depth study of Tc9 cells provides a more comprehensive understanding of the mechanism of the antitumor effect of CD8^+^ T cells.

Vδ2 T cells are the main cell population of γδ T cell subset and are a major source of IL-9 in human peripheral blood [43, 44]. In the presence of TGF-β and IL-15, Vδ2 T cells stimulated by antigens will produce a large amount of IL-9, which plays an important role in Vδ2 T cell-mediated antitumor immunity [45]. Mast cells are widely distributed around the microvasculature under the skin and visceral mucosa [46, 47], and participate in allergic diseases by secreting a variety of cytokines [48, 49]. In the intestinal mucosa, IL-9-producing mucosal mast cells (MMC9s) can promote food allergy mediated by IgE [50]. Recently, an increasing number of researchers have focused on the role of mast cells in tumorigenesis and development [51, 52]. Inhibiting mast cells activity or knocking out mast cells can eliminate tumor-specific Th9 cell-mediated antitumor effects [53–55]. In this review, we mainly summarize the different roles and underlying mechanisms of IL-9 and IL-9-producing cells (Th9 cells, Tc9 cells, Vδ2 T cells, and mast cells) in tumor immunity.

## Il-9 serves as a double-edged sword in tumor immunity

### Antitumorigenic role of IL-9

**Fig. 1:**
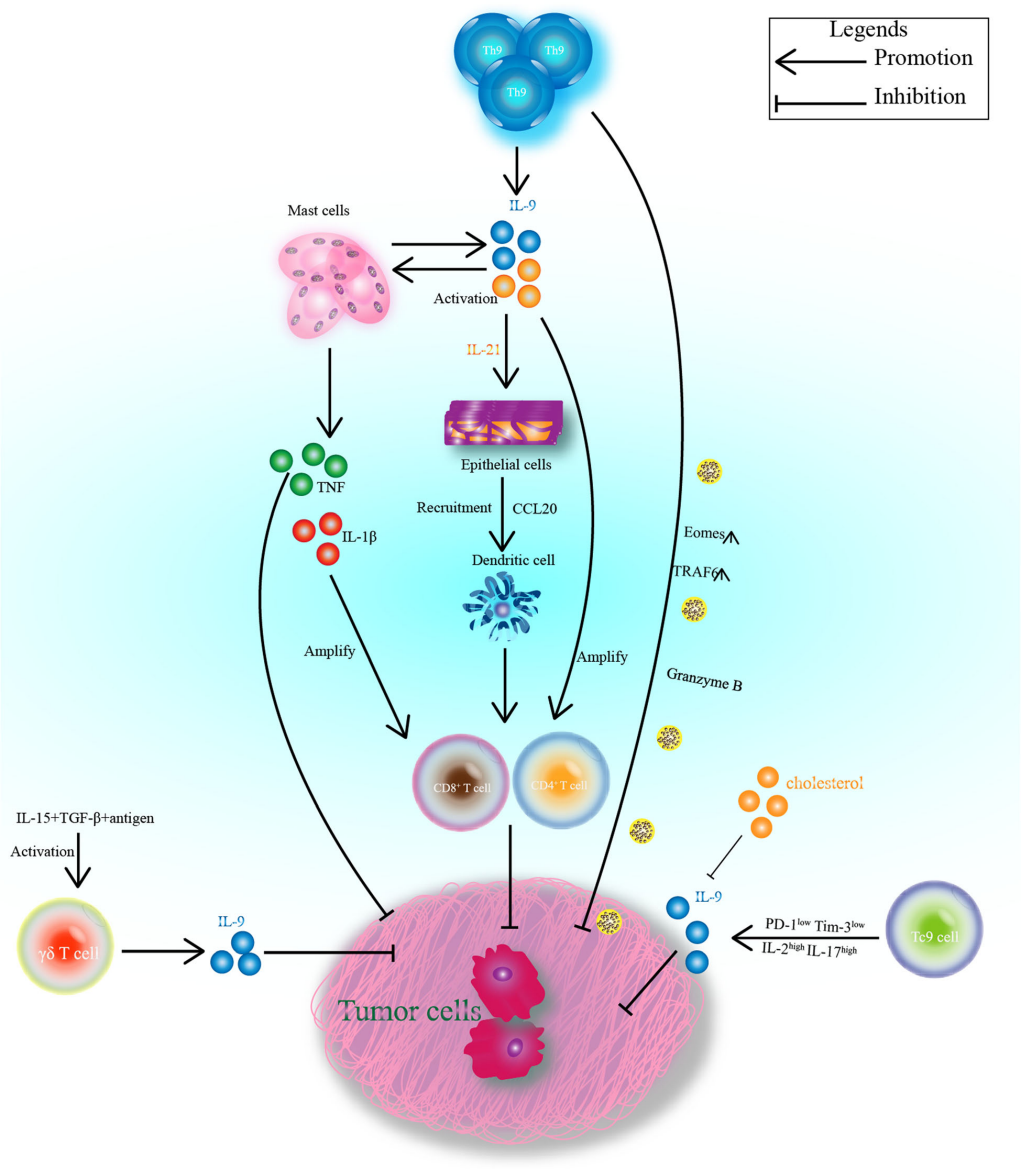
Anti-tumor effect of IL-9 and IL-9-producing cells

IL-9 can regulate antitumor immunity by activating adaptive or innate immune responses [2, 56]. In nude mice, IL-9 can significantly inhibit the growth of gastric cancer. Researchers have found that the levels of IL-4, IL-10, VEGF and TGF-β are decreased in IL-9-treated tumor-bearing nude mice. Moreover, IL-9 can inhibit the proliferation and migration of the gastric cancer cell line SGC-7901 in vitro [57]. IL-9 also exerts a strong antitumor response in colon cancer, and the expression level of IL-9 in colon cancer is positively correlated with TNM stage, lymph node metastasis, and good prognosis. In addition, IL-9 overexpression can inhibit the growth of tumors and increase the survival rate of mice with subcutaneous tumors. The antitumor effect of IL-9 may be due to the regulation of T cell function [58], the possible mechanism is shown in Fig. 1.

The antitumor effect of IL-9 is also shown in the following aspects: IL-9 can inhibit the growth of human melanoma cells; IL-9 restrains the proliferation and induces the apoptosis of HTB-72 melanoma cells by upregulating the expression of the apoptosis-promoting molecule TRAIL and the antiproliferation molecule p21 [15]; and in most of the solid tumors, IL-9 can directly promote the apoptosis of tumor cells or activate innate and adaptive antitumor immunity. In addition, the ectopically expressed membrane-bound form of IL-9 (MB-IL-9) has immune rejection and toxic effects on CT26 tumor cells and can magnify the cytotoxic effects of CD4^+^ T cells and CD8^+^ T cells [59]. Given these findings, MB-IL-9 is expected to become a potential tumor vaccine.

### Tumorigenic role of IL-9 in hematological tumors

As a lymphocyte growth factor, IL-9 can promote the proliferation and activation of lymphocytes, so in most of the hematological tumors, it exerts a tumorigenic role [60]. It has been reported that IL-9 promotes lymphoma cell survival and drug resistance in diffuse large B cell lymphoma (DLBCL). IL-9 is significantly increased in the serum of patients with DLBCL, and there is a certain correlation between IL-9 and low serum albumin and high international prognostic index scores. In vitro, IL-9 can directly induce proliferation and inhibit apoptosis in the DLBCL cell lines LY1 and LY8, promote the survival of DLBCL cells, and reduce the sensitivity of tumor cells to chemicals by upregulating the P21CIP1 gene in tumor cells [12]. Moreover, other research proves that the expression of IL-9R on DLBCL cells is significantly higher than that on normal tissues, and overexpression of IL-9R promotes the pathological process of DLBCL, which is related to poor prognosis [61]. In addition, IL-9 was significantly increased in the PBMC of chronic lymphocytic leukemia (CLL) patients compared with levels in normal individuals, and the increased IL-9 was related to the clinical staging, ZAP-70 expression, β2 microglobulin expression and IgVH status of CLL patients. Overexpression of IL-9 promotes the pathogenesis of CLL and is associated with poor prognosis [62, 63]. In cutaneous T cell lymphoma (CTCL), STAT3/5-dependent IL-9 overexpression promotes tumor cell survival. After PUUA treatment, silencing STAT5 or neutralizing IL-9 can promote tumor cell death. IL-9 knockout mice exhibit strong tumor cell growth inhibition and have more normal activated CD4^+^ and CD8^+^ T-lymphocyte production than control mice [64]. Other reports have shown that IL-9 can promote the progression of different hematological system tumors, such as Hodgkin’s lymphoma [65], acute lymphoblastic leukemia [66] and NK T cell lymphoma [67].

### Tumorigenic role of IL-9 in solid tumors

IL-9 not only exerts a tumorigenic role in the hematological tumors and in some solid tumors but also has tumor-promoting ability. It has been reported that IL-9 can promote the proliferation and migration of pancreatic cancer cells through the miR-200a/β-catenin axis. β-catenin is the target gene of miR-200a in the pancreatic cancer cells, and overexpression of miR-200a in pancreatic cancer cells can decrease the expression of β-catenin. IL-9 can reverse this phenomenon, which can reduce the expression of miR-200a on pancreatic cancer cells, thus promoting the development of pancreatic cancer [68]. Another study showed that the expression of IL-9 in colitis-associated cancer (CAC) tissue was significantly higher than that in adjacent tissues. Lentiviral vector-mediated IL-9 overexpression in the colon cancer cells lines RKO and Caco2 could promote the proliferation of colon cancer cells by upregulating the expression of c-myc and cyclin D1 [69]. In breast cancer, the level of IL-9 is increased in the serum of patients and causes tumor metastasis [70]. Dominique B et al. demonstrated that as an adaptive immune response inhibitor, IL-9 can prevent the formation of immune memory in support of tumor growth. IL-9 deficiency can make host T cells sensitive to tumors and acquire immune memory, which can effectively protect mice from the recurrence of some tumors. In their research, tumor-bearing WT and IL-9KO mice were generated, WT mice grew tumor quickly, while most IL-9KO mice had no tumorigenesis. In addition, the survival rate was higher in IL-9KO mice than in WT mice, and the tumor metastasis ability of IL-9KO mice was not as strong as that of WT mice. The results were verified by the authors in lung cancer in situ, they found that T cells were essential for tumor rejection in IL-9KO mice. In IL-9KO mice, the number of CD4^+^ and CD8^+^ T cells in the spleen and lymph nodes increased, and MDSCs decreased compared to the number in WT mice. The depletion of CD4^+^ and CD8^+^ T cells from IL-9KO mice can cause the growth of tumors. This demonstrates that IL-9 can negatively regulate T cell function and participate in tumor progression [71]. The viewpoint of Dominique Bet al. in this paper is different from common opinion of IL-9 in tumor immunity. This may be because of the complexity of the immune response, and at different stages of the disease, the same factor may have different physiological functions, which may need further research. In addition, there is evidence suggesting that IL-9 can promote the immunosuppressive function of Treg cells and protect tumor cells from the attack of immune cells [55].

**Fig. 2:**
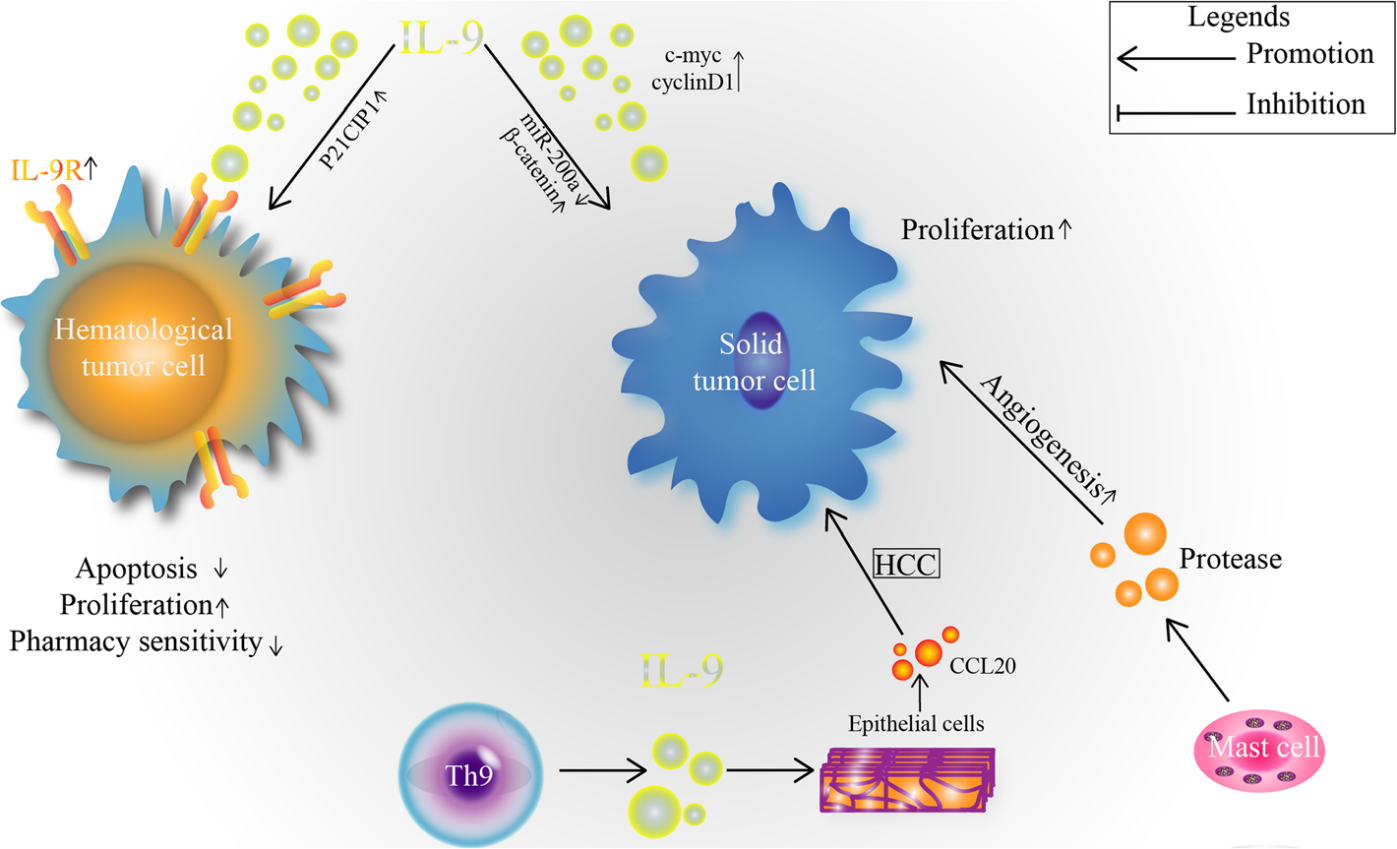
Tumorigenic role of IL-9 and IL-9-producing cells

In conclusion, IL-9 has dual roles in tumor immunity (Table 1). The main idea is that IL-9, as a lymphocyte growth factor, can promote tumor progression in hematological tumors, while in solid tumors, IL-9 can inhibit tumor development by activating innate or adaptive immune responses. However, these roles are not absolute: in some solid tumors, IL-9 also has the function of promoting tumor development (Fig. 2). Therefore, the full mechanism of IL-9 in tumor immunity needs further study.

**Table 1:** IL-9 in tumor immunity

Effect path	Effect	Target tumor	Reference
IL-10 ↑ VEGF ↑ TGF-β ↑	Inhibitor	Gastric cancer	[57]
T cell	Inhibitor	Colon cancer	[58]
CD4^+^ T CD8^+^ T cell	Inhibitor	CT26-bearing	[59]
TRAIL ↑ P21 ↓	Inhibitor	Melanoma	[15]
miR-200a ↓ β-catenin ↑	Promote	pancreatic cancer	[68]
C-myc ↑ cyclin D1 ↑	Promote	CAC	[69]
Tregs	Promote	**/**	[55]
P21CIP1 ↑	Promote	DLBCL	[12, 61]
**/**	Promote	CLL	[62, 63]
CD4^+^ T CD8^+^ T cell	Promote	CTCL	[64]
**/**	Promote	Hodgkin’s lymphoma	[65]
**/**	Promote	lymphoblastic leukemia	[66]
**/**	Promote	NK T cell lymphoma	[67]

↑: Up-regulation ↓: Down-regulation /: Not mentioned

## Different IL-9-producing cells in tumor immunity

### Antitumorigenic role of Th9

The antitumor effect of Th9 cells was first studied in melanoma. Th9 cells are the most studied IL-9-producing cells in tumor immunity, and almost all reports show that Th9 cells play an antitumor role [72, 73] (Table 2). IL-9 is the main effector cytokine by which Th9 cells exert antitumor effects, and the deletion of IL-9 will cause this effect to be lost [16]. Th9 cells promote antitumor effects mainly by activating innate immune cells and adaptive immune cells, which lead to cytotoxic effects [74]. It has been reported that increasing the numbers of Th9 cells in combination with anti-PD-1 therapy can greatly improve the clinical therapeutic effect of metastatic melanoma [73]. Th9 cells represent the third generation of T cell therapy and have high research value. Tumor-specific Th9 cells can completely cure advanced tumors, which are less exhausted, fully cytotoxic and hyperproliferative [75].

**Table 2:** IL-9-producing cells in anti-tumor immunity

Cell type	Effector factor	Pathway effect	Target tumor	Reference
Th9	IL-9	DCs ↑ → CD8^+^ T	Melanoma	[74]
Th9	/	Eomes ↑ TRAF6 ↑	Melanoma	[75]
Th9	Granzyme B	Killing effect	Melanoma	[32]
Th9	IL-9	Activate mast cell	Melanoma	[32]
Th9	IL-9 IL-21	CTL	Breast cancer	[9]
Tc9	IL-9	/	Melanoma	[76]
Tc9	IL-9	IL-7α^high^ KLRG1^low^	Breast cancer	[77, 78]
Vδ2 T	IL-9	IFN-γ ↑	/	[79, 80]
Mast cell	/	IL-1β ↑ TNF ↑	Melanoma	[31, 32, 81]

↑: Up-regulation ↓: Down-regulation /: Not mentioned →: Recruitment

Previous studies have shown that RORγ^−/−^ T cells can produce a large amount of IL-9 and strongly inhibit the growth of B16F10 melanoma. Neutralizing antibodies against IL-9 reversed this tumor-suppressive ability. Additionally, IL-9R^−/−^ mice showed increased tumor growth, and the injection of rmIL-9 into melanoma-bearing mice inhibited tumor growth. This demonstrates that IL-9 has an important antitumor role in melanoma. Animal experiments show that the growth of the tumor is greatly inhibited when tumor-specific Th9 cells are administered, and this effect can be revered by anti-IL-9. It is reported that the exogenous-IL-9 has no effect on the inhibition of melanoma in T cell-deficient mice, but mast cell deficiency will lead to the loss of the antitumor effect [32]. This suggests that Th9 cell-derived IL-9 exerts an antitumor effect mainly by activating mast cells in mouse melanoma. Moreover, IL-4 plus IL-1β can promote the differentiation of antitumor Th9 (Th9^IL-4 + IL-1β^) cells. Th9^IL-4 + IL-1β^ cells are a distinct T cell subset that can produce IL-9 in a manner that is dependent on the NF-κB signaling pathway, and the inhibition of NF-κB will lead to blockade of IL-9 production. Compared to traditional Th9 (Th9^IL-4 + TGF-β^) cells, Th9^IL-4 + IL-1β^ cells show stronger cytotoxic effect and tumor killing ability, exerting a powerful antitumor effect in melanoma [24]. In addition, tumor-specific Th9 cells can eliminate advanced tumors, which depends on IL-9 and upregulated expression of Eomes and TRAF6. To prove this idea, OVA-specific CD45.1^+^ OTII Th1, Th2 and Th17 cells were adoptively transferred into B16 tumor-bearing mice, and the data showed that only Th9 cells could mediate tumor regression and prolong the survival rate of mice, while Th1 and Th17 cells only induced transient tumor regression, and the tumor would recur. Therefore, Th9 cells have a stronger and longer antitumor ability than Th1 and Th17 cells [75]. In addition to their role in the melanoma, Th9 cells can play an antitumor role in breast cancer via IL-9 and IL-21. In the circulation of breast cancer patients, Th9 cells were significantly increased compared to the levels in healthy people. The increased Th9 cells mainly belong to the CCR4^−^ CCR6^−^ CXCR3^−^ subset and can secrete IL-9 and IL-21 after TCR stimulation. IL-9 and IL-21 can amplify the cytotoxic effect of CD8^+^ T cells, thus promoting an antitumor effect in breast cancer [9]. This is contradictory to the result we aforementioned result and such contradictory result is common because breast cancer in human and mice may have different mechanisms, which need to be studied further. Moreover, Th9 cells and Th9 cell-derived IL-9 also have a strong ability to inhibit tumor metastasis and growth in metastatic lung cancer and gastric cancer in nude mice [57, 70]. Th9 cells can also kill tumor cells directly by secreting granzyme B in some tumors [32].

### Different factors regulate the antitumor effect of Th9 cells

**Fig. 3:**
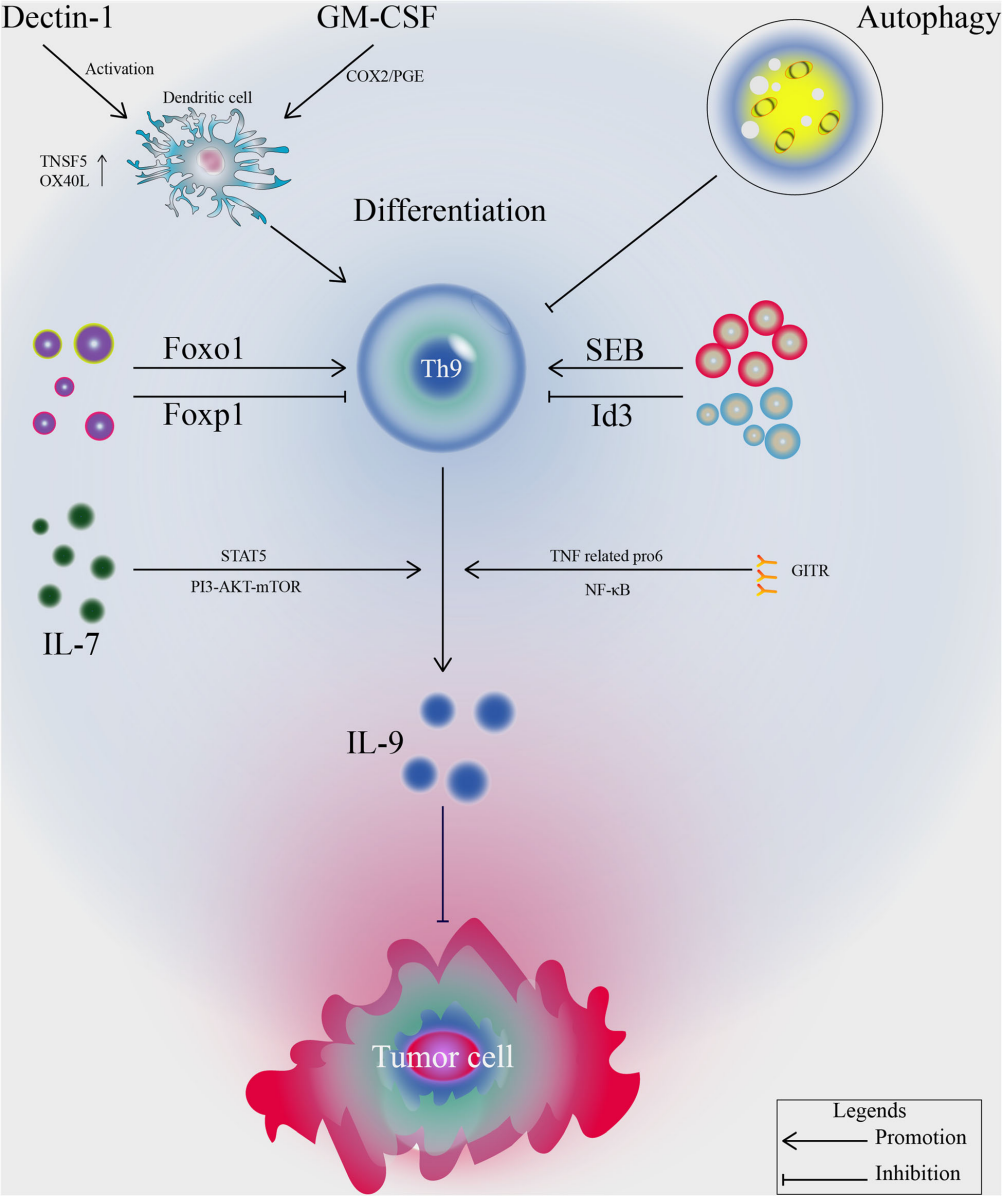
Different factors regulate anti-tumor effect of Th9 cells

There are many factors that regulate the function and differentiation of Th9 cells to participate in tumor immunity (Fig. 3). It has been reported that DCs activated by Dectin-1 agonists can upregulate TNFSF15 and OX40L, which can promote the differentiation of Th9 cells and participate in the antitumor response [82]. Glucocorticoid-induced tumor necrosis factor receptor (GITR) can promote tumor regression by inducing the production of IL-9-producing CD4^+^ T cells. Moreover, GITR can enhance the differentiation of Th9 cells in a TNFR-related protein 6- and NF-κB-dependent manner and inhibit the production of Treg cells [83]. Increased IL-9 promotes tumor-specific cytotoxic T lymphocytes and tumor regression by enhancing the function of DCs. GM-CSF can also activate DCs and induce tumor-specific Th9 cells differentiation through the COX2/PGE signaling pathway [84]. Autophagy plays an important role in anti-tumor effects by selecting inhibiting the differentiation of Th9 cells. CD4^+^ T cell deficiency of the autophagy genes Atg3 and Atg5 can enhance the differentiation of Th9 cells and the expression of IL-9. Therefore, blocking autophagy can enhance the differentiation of Th9 cells and promote antitumor effects [84]. In addition, IL-7 can promote histone acetylation at the promoter of the IL-9 gene by activating the STAT5 and PI3K-Akt-mTOR signaling pathways and increasing the expression of IL-9. Foxo1 and Foxp1 play opposite roles in regulating Th9 cell differentiation and antitumor activity. Foxo1 induces the differentiation of IL-9-derived Th9 cells, while Foxp1 plays an inhibitory role in Th9 cells differentiation, providing a promising target for anti-tumor immunotherapy [85].

The DNA-binding inhibitor Id3 can regulate the differentiation of Th9 cells, and deletion of Id3 can increase the production of IL-9 in CD4^+^ T cells. The mechanism is that TGF-β and IL-4 downregulate the expression of Id3 in the presence of TAK1 kinase. Downregulated Id3 then promotes the binding of the transcription factors E2A and GATA3 to the promoter region of the IL-9 gene, thus promoting the transcription of IL-9. Research has shown that Id3 can regulate the antitumor immunity of experimental melanoma-bearing mice by negatively regulating the differentiation of Th9 cells [86]. A metabolite of a bacterium, staphylococcal enterotoxin B (SEB) can induce the proliferation of Th9 cells and inhibit the growth of mouse squamous carcinoma [87]. It has also been reported that SEB can promote the induction of specific Th9 cells to inhibit glioma cell growth [88] (Table 3).

**Table 3:** Regulatory factors of IL-9-producing cells in tumor immunity

Regulatory factor	Regulatory pathway way	Aim cell	Target tumor	Reference
Dectin-1 activated DCs	TNFSF15 ↑ OX40L ↑	Th9 ↑	Melanoma	[82]
GITR	TNFR-ralated Pr 6 NF-κB	Th9 ↑	CT26-bearing Melanoma	[83]
Autophage	Atg3 Atg5	Th9 ↓	/	[84]
IL-7	STAT5 PI3K-Akt-mTOR	Th9 ↑	Melanoma	[85]
Foxo1	/	Th9 ↑	Melanoma	[85]
Foxp1	/	Th9 ↓	Melanoma	[85]
Id3	/	Th9 ↓	melanoma	[86]
SEB	HDAC1 ↑ PU.1 ↑	Th9 ↑	SqC	[87]
GM-CSF	COX2/PGE	Th9 ↑	/	[84]
Cholesterol	LXR P65	Tc9 ↓	MC38-gp100-bearing	[42]
TGF-β IL-15	/	Vδ2 T ↑	/	[80]
IL-9	/	Mast cell ↑	melanoma	[31, 32, 81]

↑: Up-regulation or activation ↓: Down-regulation or depression /: Not mentioned

### Tumorigenic role of Th9 cells

Only one report has proven that Th9 cells can serve a tumor-promoting role in human hepatocellular carcinoma (HCC) via the CCL20 and STST3 signaling pathways. The number of IL-9-producing Th9 cells is significantly increased in the circulation and tumor tissue of HCC patients, and the more Th9 cells there are, the shorter the survival rate of HCC patients. Previous studies have shown that CCL20 induces epithelial-stromal changes and is related to poor prognosis of in HCC. Coculture of HCC cells with autologous Th9 cells results in upregulation of CCL20 expression on tumor cells in vitro. The main reason for the tumorigenic role of Th9 cells is that Th9 cell-derived IL-9 can promote the expression of CCL20 by activating the STAT3 signaling pathway [89] (Fig. 2).

In general, as an important source of IL-9, Th9 cells play an important role in antitumor immunity. In the tumor microenvironment, many factors can positively or negatively regulate the antitumor effect of Th9 cells. They are expected to be the target of immunotherapy in some tumors due to their strong and long-lasting anti-tumor effect.

### Tc9 cells in tumor immunity

Tc9 cells are a subset of IL-9-producing CD8^+^ T cells and can be induced in Th9 cell differentiation conditions by CD8^+^ T cells [25, 38, 90]. Recent studies have shown that Tc9 cells have a stronger antitumor effect than conventional IFN-γ-producing CD8^+^ T cells. To prove the antitumor effect of Tc9 cells, in the experiment of Lou Y et al., Tc1 and Tc9 cells were adoptively transferred into B16-OVA-bearing CD45.1-transgenic mice with a DC vaccine and rmIL-2. Their results showed that Tc9 cells could mediate complete tumor regression and long-term survival, while the mice that received Tc1 cells relapsed in the fourth week [76]. The reason is that the percentage and number of CD8^+^ T cells in the spleens of mice that received Tc9 cells was more than that in the spleens of mice that received Tc1 cells, and the number of Annexin V^+^ cells in Tc1 cell-transferred mice was greater than that in Tc9-transferred mice. More importantly, Tc9 cells are less exhausted and can develop into fully functional cells in vivo. Transferred Tc1 cells can become transient effector cells of terminal differentiation with KLRG1^high^ and IL-7α^low^ phenotypes [77]. However, transferred Tc9 cells highly expressed IL-7α, which is a prosurvival cytokine receptor, indicating that Tc9 cells can promote a sustained antitumor effect in melanoma [38]. Moreover, when stimulated with PMA or anti-TCR antibodies, CD8^+^ T cells can shift toward Tc9 cells in breast cancer. IL-9-producing CD8^+^ T cells highly express IL-2 and IL-17 in breast cancer and have low expression of the inhibitory receptors PD-1, KLRG1 and Tim-3. Additionally, exogenous IL-9 can promote the expression of IL-9 and IL-9R on IL-9R^high^ CD8^+^ T cells [78]. Similar to Th9 cells, Tc9 cells also can be regulated by some factors in the tumor microenvironment. IL-9 is essential for the continuous antitumor effect of Tc9 cells, and cholesterol can induce LXR Quasi ubiquitination, and reduce the binding of p65 to the *il9* promoter, resulting in a decrease in IL-9 secretion, thus affecting the antitumor effect of Tc9 cells [42].

### Vδ2 T cells in tumor immunity

Vδ2 T cells are one of the major subsets of γδ T cells, a major source of IL-9 in human peripheral blood [91]. At present, there are few studies on Vδ2 T cell-derived IL-9 in diseases. One report demonstrated that Vδ2 T cell-derived IL-9 was involved in the pathological process of Schistosoma japonicum infection [92]. Christian Peters et al. suggested that the adoptive transferred of IL-9-producing Vδ2 T cells into cancer patients may become a new strategy for tumor treatment [79]. According to other reports, the Vδ2 T cells have cytotoxic effects on tumor cells by secreting IFN-γ. In the presence of TGF-β and IL-15, Vδ2 T cells stimulated by antigens can produce a large amount of IL-9, which has a strong antitumor effect in some solid tumors [80]. Presumably, Vδ2 T cell-derived IL-9 may play a vital role in the Vδ2 T cell-mediated antitumor effect [79]. This result complements the antitumor mechanism of Vδ2 T cells and provides a new idea for Vδ2 T cell-mediated antitumor immune therapy.

### Mast cells in tumor immunity

Mast cells are involved in the pathological process of various allergic diseases [48], autoimmune diseases [93] and cancers [94]. Previous studies have shown that mast cell-derived IL-9 can promote susceptibility to IgE-mediated experimental food allergy [50]. In a variety of cancers, the more mast cells that had infiltrated into tumor tissue, the higher the survival rate of patients was [55]. Th9 cell-derived IL-9 can activate mast cells to secrete IL-2, IL-1β and TNF. Secreted IL-1β and IL-2 can enhance the differentiation of Th9 cells, and TNF will cause tumor cells to die [31, 32, 81]. In addition, mast cells themselves can also secrete IL-9 to enhance antitumor immunity [50]. In addition to the antitumor effect, mast cells can also serve a tumorigenic role in tumor immunity. The protease released by activated mast cells is the key factor in inducing angiogenesis, which may promote tumor angiogenesis and tumor growth [95]. Moreover, it has been reported that mast cells promote small bowel cancer progression in a tumor stage-specific and cytokine-dependent manner [96]. The role of IL-9 and IL-9 producing cells in antitumor immunity is summarized in Fig. 1 and Table 2.

## Conclusions

As a pleiotropic cytokine, IL-9 can participate in the progression of various diseases. Recently, the roles of IL-9- and IL-9-producing cells in tumor immunity have attracted increasing attention. IL-9- and IL-9-producing cells have dual roles in tumor immunity. Although most of the current studies focus on the antitumor effect of IL-9- and IL-9-producing cells, their tumorigenic effect cannot be ignored. IL-9 in solid tumor cells always inhibits proliferation and promotes apoptosis of tumor cells. As a lymphocyte growth factor, IL-9 can promote the deterioration of hematological neoplasms, and it is worth noting that IL-9 can also promote the growth of some solid tumors, such as pancreatic cancer and CAC. More importantly, the inhibitory effect of IL-9 on the adaptive immune response can destroy the sensitivity of host T cells to tumor cells. Th9 cells are a newly identified CD4^+^ T cell subset that can activate innate and adaptive immune responses by secreting IL-9 and IL-21. The antitumor effect of Th9 cells was first studied in melanoma. Moreover, Th9 cells can also secrete granzyme B and directly participate in the killing of tumor cells, but only one report has proven that Th9 cells exert a tumorigenic role in human hepatocellular carcinoma (HCC). In the tumor microenvironment, many factors can regulate the antitumor effect of Th9 cells. Tc9 cells can be induced under conditions that lead to Th9 cell differentiation. Tc9 cells are involved in antitumor activity via cytotoxic effects and the secretion of IL-9. Compared with the traditional IFN-γ-secreting Tc1 cells, Tc9 cells have a stronger and more lasting antitumor effect in OT-I/B16-OVA and pmel-1/B16 mouse melanoma models. The study of Tc9 cells in tumor immunity has highlighted the tumor-killing function of traditional CD8^+^ T cells. Vδ2 T cells, one of the major subsets of γδT cells, exert antitumor effects by secreting IL-9. Mast cells can not only be activated by IL-9, but also secrete IL-9 to promote the activation of T cells involved in regulating the antitumor immunity. Mast cells can promote tumor processes by secreting tumor angiogenic factors. Many other cells can secrete IL-9, such as NKT cells and ILC2s, but their role in tumor immunity is unclear [97, 98]. Further studies are warranted to obtain a comprehensive understanding of IL-9 and IL-9-producing cells in tumor immunity, which may contribute to finding more suitable targets for the clinical treatment of cancers.

## Data Availability

Not applicable.

## Abbreviations

IL-9: Interleukin-9
Th9: T-helper 9
Tc9: IL-9-producing CD8^+^ T
IL-9R: IL-9 receptor
ILC2s: Type 2 innate lymphoid cells
DCs: Dendritic cells
MMC9s: IL-9-producing mucosal mast cells
MB-IL-9: Membrane-bound form of IL-9
DLBCL: Diffuse large B cell lymphoma
PBMC: Peripheral blood mononuclear cells
CLL: Chronic lymphocytic leukemia
CTCL: Cutaneous T cell-lymphoma
CAC: Colitis-associated cancer
MDSCs: Myeloid-derived suppressor cells
GITR: Glucocorticoid-induced tumor necrosis factor receptor
SEB: Staphylococcal enterotoxin B
HCC: Hepatocellular carcinoma
